# c-Met and EPHA7 Receptor Tyrosine Kinases Are Related to Prognosis in Clear Cell Renal Cell Carcinoma: Focusing on the Association with Myoferlin Expression

**DOI:** 10.3390/cancers14041095

**Published:** 2022-02-21

**Authors:** Minsun Jung, Seokhyeon Lee, Kyung Chul Moon

**Affiliations:** 1 Department of Pathology, Severance Hospital, Yonsei University College of Medicine, Seoul 03722, Korea; jjunglammy@yuhs.ac; 2 Department of Pathology, Seoul National University College of Medicine, Seoul 03080, Korea; lshlove1023@gmail.com; 3 Kidney Research Institute, Medical Research Center, Seoul National University College of Medicine, Seoul 03080, Korea

**Keywords:** clear cell renal cell carcinoma, MET receptor tyrosine kinase, myoferlin, prognosis, immunohistochemistry

## Abstract

**Simple Summary:**

Receptor tyrosine kinases are essential for the development, growth, and progression of clear cell renal cell carcinoma (ccRCC). Targeted therapies using receptor tyrosine kinase inhibitors are widely used in ccRCC treatment. Myoferlin is a well-known protein that regulates various receptor tyrosine kinases. We aimed to identify myoferlin-associated receptor tyrosine kinases and their prognostic implications in ccRCC. After screening with proteomic analysis, we focused on c-Met and EPHA7 receptor tyrosine kinases. c-Met expression was associated with poor prognosis in ccRCC, and there was an indication that the c-Met pathway may be regulated by myoferlin. Although the expression of EPHA7 and myoferlin was not corelated, the expression of EPHA7 was also associated with unfavorable outcomes. These results suggest that c-Met and EPHA7 might be useful prognostic biomarkers, and the presumed myoferlin/c-Met pathway could be a novel therapeutic target in ccRCC.

**Abstract:**

Receptor tyrosine kinases (RTKs) are important targets for clear cell renal cell carcinoma (ccRCC) treatment. Myoferlin is a strong regulator of RTKs. To identify myoferlin-associated RTKs and their prognostic implications in ccRCC, we investigated the expression of RTKs and myoferlin using proteome-based evaluation and immunohistochemical staining in tissue microarray. Multivariate Cox analysis adjusted for TNM stage and WHO grade was performed (*n* = 410 and 506). Proteomic analysis suggested c-Met and EPHA7 as novel candidates for myoferlin-associated RTKs. We immunohistochemically validated the positive association between c-Met and myoferlin expression. High c-Met expression was independently associated with overall (hazard ratio (HR) = 1.153–2.919) and cancer-specific survival (HR = 1.150–3.389). The prognostic effect of high c-Met expression was also determined in an independent cohort (overall survival, HR = 1.503–3.771). Although expression of EPHA7 and myoferlin was not correlated, EPHA7 expression was independently associated with progression-free (HR = 1.237–4.319) and cancer-specific survival (HR = 1.214–4.558). In addition, network-based prioritization showed co-functional enrichment of c-Met and myoferlin, suggesting a novel regulatory function of myoferlin in c-Met signaling. This study indicates that c-Met and EPHA7 might be useful prognostic biomarkers, and the presumed myoferlin/c-Met pathway could be a novel therapeutic target in ccRCC.

## 1. Introduction

Receptor tyrosine kinases (RTKs) are essential for the development, growth, and progression of clear cell renal cell carcinomas (ccRCCs) [1]. Some RTKs that are frequently dysregulated in ccRCC include vascular endothelial growth factor receptor (VEGFR), c-Met, epidermal growth factor receptor (EGFR), and human epidermal growth factor receptor-2 (HER2). These RTKs initiate downstream signaling pathways, such as PI3K/AKT/mTOR, phospholipase C, and RAS/MAPK/RAF/ERK [2,3]. The dependence of RTKs in ccRCC pathobiology has led to biomarker-driven targeted therapies in ccRCC and has resulted in the Food and Drug Administration and European Medicines Agency approval of tyrosine kinase inhibitors, including sunitinib, axitinib, and cabozantinib, as first-line or second-line regimens, [4]. In addition, several clinical trials are currently underway, either for the extended usage of existing RTK inhibitors or for the study of new inhibitors, in renal cell carcinomas (Table S1) [3].

Myoferlin is a well-known transmembrane protein that affects the expression, function, and stability of several RTKs, including VEGFR2, along with its ligand vascular endothelial growth factor-A (VEGFA) [5,6], TEK [7], insulin-like growth factor 1 receptor [8], and EGFR [9,10,11]. Previous studies have presented various functions of myoferlin in the regulation of RTKs, which appear to be dependent on the cell type and context [5,7,8,9,10,11,12]. Understanding myoferlin-mediated RTK regulation may help to control the complex network of RTKs and their crosstalk and prevent treatment resistance to RTK-targeted therapeutics in ccRCCs [13]. Therefore, comprehensive identification of the connection between RTKs and myoferlin is important for better utilization of RTKs as prognostic and therapeutic biomarkers in ccRCC.

In this study, we aimed to identify myoferlin-associated RTKs and their prognostic implications in ccRCC. To this end, we comprehensively evaluated the correlation between RTKs and myoferlin in ccRCC by analyzing liquid chromatography with tandem mass spectrometry (LC–MS/MS) proteomic data taken from the Clinical Proteomic Tumor Analysis Consortium (CPTAC) [14]. The results were validated using immunohistochemical (IHC) staining, and the prognostic implications of RTK expression were determined in an independent cohort. We also analyzed the functional interaction of myoferlin and c-Met in ccRCC using a co-functional network model.

## 2. Materials and Methods

### 2.1. Identification of Receptor Tyrosine Kinases Involved in Clear Cell Renal Cell Carcinoma Using Proteomic Dataset

Normalized LC–MS/MS proteome data of the RTK family and myoferlin were obtained from the CPTAC portal, which included 103 microscopically and molecularly confirmed ccRCCs [14]. A comprehensive list of RTKs was collected from the HUGO Gene Nomenclature Committee database (https://www.genenames.org/; accessed on 30 October 2019) [15]. The correlations between myoferlin and the RTK family were assessed.

### 2.2. Immunohistochemical Staining for Receptor Tyrosine Kinases and Ligands

We included 410 patients with ccRCC who underwent partial or radical nephrectomy at Seoul National University Hospital between 2005 and 2008. An independent cohort of 506 patients with ccRCC (2009–2011) was also included to validate the prognostic effect of c-Met expression. Double 2 mm cores were obtained from formalin-fixed paraffin-embedded tissues for tissue microarray (TMA) construction. Samples that had been collected from patients that had received neoadjuvant treatment, presented with bilateral disease at the time of diagnosis, or had Von Hippel–Lindau syndrome were excluded from the study. The RTK ligands were selected based on the literature search [3]. The following RTKs and ligands were examined using IHC staining: c-Met (ready-to-use, SP44, Ventana, Tucson, AZ, USA), Eph receptor A7 (EPHA7; 1:800, 6C8G7, Novus Biologicals, Centennial, CO, USA), HER2 (ready-to-use, 4B5, Ventana), hepatocyte growth factor (HGF; 1:100, ab118871, Abcam, Cambridge, UK), VEGFA (1:300, sc-7261, Santa Cruz Biotechnology, Dallas, TX, USA), and ephrin A5 (EFNA5; 1:100, LS-C356004, Lifespan Biosciences, Seattle, WA, USA). IHC staining was conducted with a Benchmark autostainer (Ventana), according to the manufacturer’s instructions, using 4 µm thick TMA slides. The expression of RTKs and ligands in the membranes of tumor cells found in the TMA cores was measured as the sum of staining proportion (0: absent; 1: 0–1%; 2: 1–10%; 3: 10–33%; 4: 33–66%; and 5: 67–100%) and predominant intensity (0: absent; 1: weak; 2: moderate; and 3: strong) [16,17]. In the discordant cases, an average was adopted. The expression of myoferlin was retrieved from a previously published work [11], where expression was recorded as high if more than 1/3 of tumor cells were stained intensely or more than 2/3 of tumor cells were stained lightly in the cell membrane.

### 2.3. Clinical Information and International Metastatic RCC Database Consortium Risk Groups

Clinical data regarding lifestyle factors and International Metastatic RCC Database Consortium (IMDC) scores were collected from medical records. Body mass index (BMI) was calculated by dividing body weight (kg) by height squared (m^2^). According to the Korean Alcohol Guidelines for Moderate Drinking [18], heavy drinking was defined as >8 drinks/week for men and >4 drinks/week for women. The IMDC risk score was developed to stratify the survival of metastatic renal cell carcinomas based on six adverse factors (low Karnofsky performance status (KPS), early systemic therapy, high calcium, low hemoglobin, high neutrophils, and high platelets) [19]; no adverse factors indicated favorable risk, one or two adverse factors indicated intermediate risk, and three or more indicated poor risk. Because KPS was not available for our patients, the good/intermediate risk group and the poor risk group without a KPS score were determined with IMDC scores 0/1 and 3, respectively.

### 2.4. Survival Analysis Using Immunohistochemical Expression

We compared progression-free survival (PFS), overall survival (OS), and cancer-specific survival (CSS) according to high or low IHC expression using Kaplan–Meier curves and log-rank tests. The optimal cutoff value of high RTK expression was determined based on the statistical significance in the survival analysis, resulting in scores of ≥6.5 for c-Met and ≥5.5 for EPHA7. PFS, defined as the interval between surgery and locoregional recurrence or distant metastasis, was determined according to the Response Evaluation Criteria in Solid Tumors (RECIST) 1.1 criteria [20]. OS was calculated as the time between surgery and death. CSS was defined as the interval between surgery and cancer-related death. The prognostic effects of IHC expression were assessed using univariate and multivariate Cox proportional hazard models.

### 2.5. Network-Based Prioritization of Interacting Proteins and Functional Enrichment Analysis

We developed an interacting protein set for c-Met and myoferlin by reconstructing a combined network of proteins prioritized from STRING (ver. 11.5) [21], and GeneMANIA (ver. 3.5.2) [22]. To maximize the discovery of the shared functional domain of c-Met and myoferlin, we included molecules that were commonly neighboring c-Met and myoferlin. The selected proteins (nodes) and interaction scores (edges) were visualized using Cytoscape (ver. 3.8.2) [23]. Gene ontology biological process (GOBP) enrichment of the gene set was investigated using ToppGene Suite at a significance level of false-discovery rate < 0.05 [24].

### 2.6. Statistical Analysis

The χ2 test was performed for the clinicopathological variables. Pearson’s correlation coefficient was calculated for proteome abundance, with a prior normality assumption (Kolmogorov–Smirnov *p* > 0.05). IHC expression scores were compared nonparametrically. Statistical analyses were performed using R software (ver. 3.6.3), with a two-tailed *p*-value < 0.05 being considered as significant.

## 3. Results

### 3.1. Receptor Tyrosine Kinases Correlated with Myoferlin in the Proteomic Dataset

**Figure 1:**
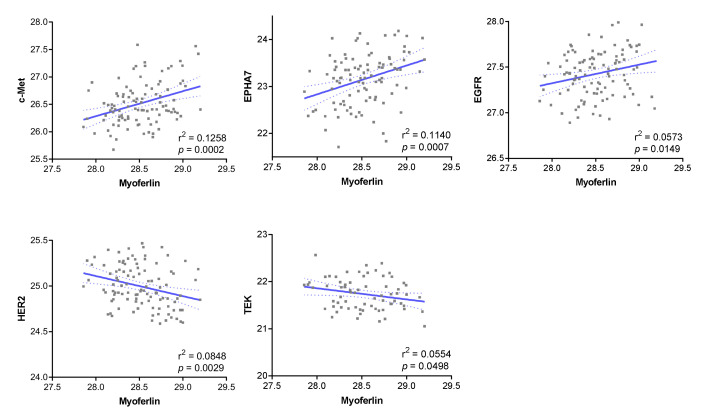
Receptor tyrosine kinase proteins identified in the clear cell renal cell carcinoma proteomic dataset and their correlation with myoferlin. Dotted lines denote the 95% confidence interval of Pearson’s correlation.

In the ccRCC proteomic dataset, we identified 38 RTKs at a range of variable frequency (6.8–100% of 103 cancers) (Table S2), while myoferlin was expressed in all samples. Among the RTKs, c-Met (*p* = 0.0002), EPHA7 (*p* = 0.0007), and EGFR (*p* = 0.0149) were positively correlated with myoferlin expression, whereas HER2 (*p* = 0.0029) and TEK (*p* = 0.0498) were negatively correlated (Figure 1). We further investigated Ki-67, p53, CXCR4, CA9, and VHL, which are important non-RTK proteins in ccRCC and other cancers. There was no association between myoferlin and these proteins (Figure S1).

Among the five RTKs that correlated with myoferlin, EGFR, which has previously been verified using IHC staining [11], and TEK, which showed a relatively low frequency (68% in the proteomic data) and loose correlation with myoferlin (Pearson’s r^2^ = 0.0554, *p* = 0.0498) (Table S2), were excluded from further validation. HER2 was also subsequently excluded because of a rare immunoreaction (1.5%, 3/202) in a pilot study. Therefore, the associations between c-Met and EPHA7 with myoferlin expression were investigated using IHC staining, and the prognostic implications were examined.

### 3.2. c-Met Expression Was Significantly Related to Myoferlin Expression and Pathological Parameters in ccRCC

IHC staining for c-Met was analyzed in 410 ccRCC specimens using TMA slides. The male to female ratio was 2.8:1, and the median age was 58.5 years (ranging between 20 and 81). Among the patients, 272 (66.3%) underwent radical nephrectomy, while the others (33.7%) underwent partial excision. There were 90 patients (22.0%) that were in TNM stage III or IV, and 217 (52.9%) that were in WHO grade 3 or 4. Lifestyle factors, including BMI, smoking status, and alcohol use, were available for 360 patients. The median BMI was 24.4 kg/m^2^ (ranging between 22.7 and 26.4), 67 patients (18.6%) were current or ex-smokers, and 39 (10.8%) were heavy drinkers (Table 1). The median follow-up period was 121 months.

**Figure 2:**
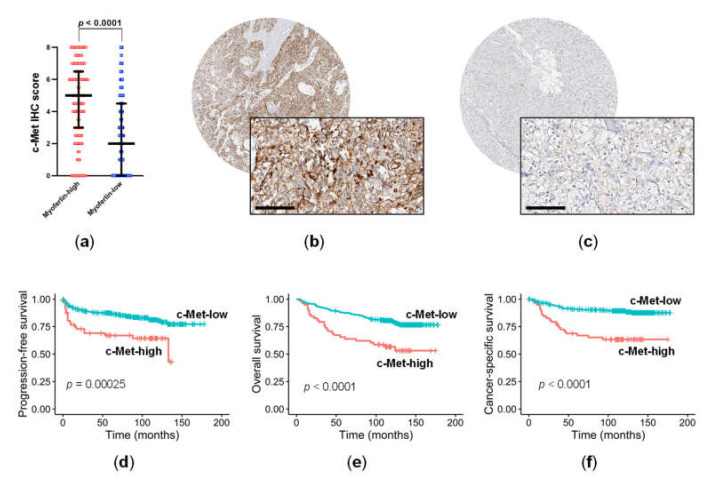
Immunohistochemical (IHC) staining for c-Met in relation to myoferlin expression and prognosis in clear cell renal cell carcinoma. (**a**) The IHC staining level of c-Met was significantly higher in cancers with high expression of myoferlin than in those with low myoferlin expression, (**b**) high c-Met expression (score 8, strong expression in 67–100%), (**c**) low c-Met expression (score 3, weak expression in 1–10%), (**d**) progression-free survival, (**e**) overall survival, and (**f**) cancer-specific survival. Scale bar = 100 μm.

IHC staining for c-Met was prominent in the cell membrane. Consistent with the proteomics results, c-Met was significantly upregulated in ccRCC samples that had high myoferlin expression compared to those with low myoferlin expression (Mann–Whitney test, *p* < 0.0001) (Figure 2a). For survival analysis, the expression of c-Met was classified as high in 58 (14.1%) (Figure 2b), and low in 352 samples (85.9%) (Figure 2c). Kaplan–Meier curves with log-rank tests showed that high c-Met expression was associated with adverse PFS (*p* = 0.00025; Figure 2d), OS (*p* < 0.0001; Figure 2e), and CSS (*p* < 0.0001; Figure 2f). In line with the different survival rates, high c-Met expression correlated with the poor IMDC risk group (*p* = 0.0058), high TNM stage (III/IV vs. I/II, *p* = 0.0078), and high WHO grade (3/4 vs. 1/2, *p* < 0.0001) in clinicopathological data (Table 1). High c-Met expression was also associated with high myoferlin expression (*p* < 0.0001) (Table 1), and a previous study showed that this was related to an unfavorable prognosis in ccRCC [11].

### 3.3. High c-Met Expression Was an Independent Negative Prognostic Factor in ccRCC

The effects of high c-Met expression on ccRCC prognosis was assessed using Cox regression analysis. Univariate analysis showed a significant association between high c-Met expression and short PFS (hazard ratio (HR) = 2.516, 95% confidence interval (CI) = 1.514–4.183, *p* = 0.0004), OS (HR = 2.581, 95% CI = 1.652–4.032, *p* < 0.0001), and CSS (HR = 3.811, 95% CI = 2.239–6.485, *p* < 0.0001) (Table 2). Multivariate analysis selected high c-Met expression as an independent prognostic factor for shorter OS (adjusted HR = 1.834, 95% CI = 1.153–2.919; *p* = 0.0105) and CSS (adjusted HR = 1.974, 95% CI = 1.150–3.389; *p* = 0.0137) when adjusted for TNM stage (III/IV vs. I/II) and WHO grade (3/4 vs. 1/2) (Table 2). TNM stage and WHO grade were also independently associated with all the prognostic endpoints (Table 2).

The prognostic effect of c-Met expression was also determined in an independent cohort (*n* = 506). The male to female ratio was 2.8:1, and the median age was 56.5 years (ranging between 19 and 87). There were 93 patients (18.4%) that were in TNM stage III or IV, and 196 (38.7%) that were in WHO grade 3 or 4. The median follow-up period was 126 months. The expression of c-Met was classified similarly, as high in 49 (9.7%) and low in 457 (90.3%). Multivariate Cox regression analysis adjusted for TNM stage (III/IV vs. I/II) and WHO grade (3/4 vs. 1/2) showed that high c-Met expression was an independent, negative prognostic factor for OS (adjusted HR = 2.381, 95% CI = 1.503–3.771; *p* = 0.0002) (Table 3).

### 3.4. EPHA7 Expression Was Not Correlated with Myoferlin Expression, but Was Independently Associated with Prognosis in ccRCC

**Figure 3:**
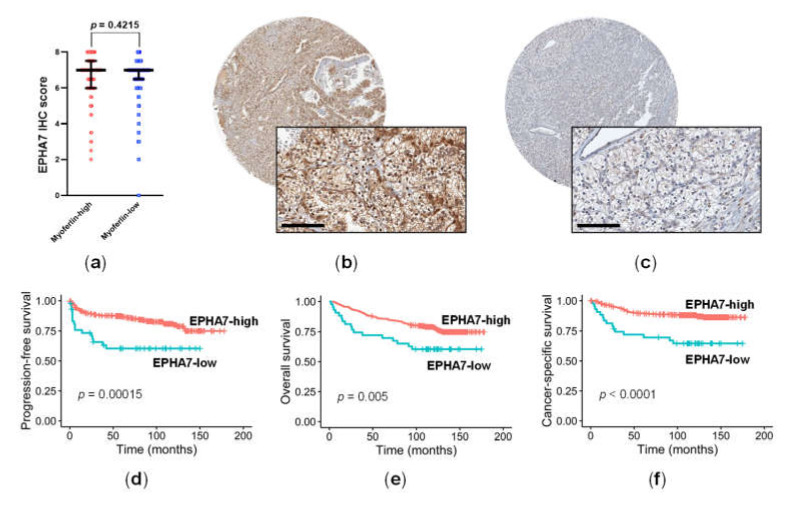
Immunohistochemical (IHC) staining for EPHA7 in relation to myoferlin expression and prognosis in clear cell renal cell carcinoma. (**a**) The IHC staining level of EPHA7 was similar between myoferlin-high and myoferlin-low cancers, (**b**) high EPHA7 expression (score 8, strong expression in 67–100%), (**c**) low EPHA7 expression (score 2, weak expression in 0–1%), (**d**) progression-free survival, (**e**) overall survival, and (**f**) cancer-specific survival. Scale bar = 100 μm.

Next, the expression levels of IHC staining for EPHA7 were determined in the primary cohort. EPHA7 staining was available in 408 ccRCCs, and this was positive in the cell membrane and cytoplasm. Although we failed to find an association between EPHA7 and myoferlin expression (Mann–Whitney test, *p* = 0.4215; Figure 3a), we found a correlation between EPHA7 expression, which was high in 375 (91.9%) patients and low in 33 (8.1%) (Figure 3b,c), and patient prognosis. Low EPHA7 expression was significantly associated with shorter PFS (*p* = 0.00015; Figure 3d), OS (*p* = 0.005; Figure 3e), and CSS (*p* < 0.0001; Figure 3f) in patients with ccRCC. In addition, low EPHA7 expression had a negative impact on PFS (HR = 2.551, 95% CI = 1.358–4.644, *p* = 0.0033) and CSS (HR = 2.979, 95% CI = 1.548–5.733, *p* = 0.0011). When adjusted for TNM stage (III/IV vs. I/II) and WHO grade (3/4 vs. 1/2), low EPHA7 expression was selected as an independent, unfavorable prognostic factor for PFS (adjusted HR = 2.311, 95% CI = 1.237–4.319; *p* = 0.0086) and CSS (adjusted HR = 2.352, 95% CI = 1.214–4.558; *p* = 0.0113) (Table 4).

### 3.5. Network-Based Prioritization of Interacting Proteins for c-Met and Myoferlin

**Figure 4:**
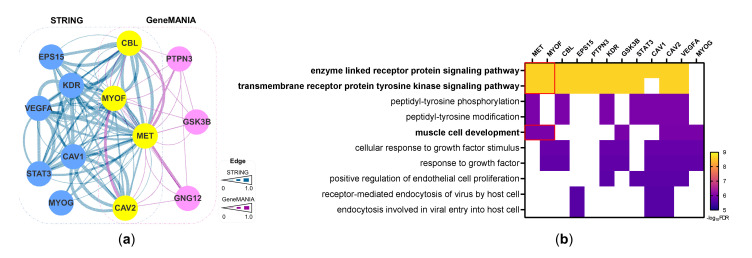
Network-based co-functional model of c-Met and myoferlin. (**a**) The 13 interacting proteins centered on c-Met and myoferlin. The blue and pink nodes are proteins identified in STRING and GeneMANIA datasets, respectively, and the yellow nodes are proteins that are common in both datasets. The edges are connections between proteins, ranging from 0 to 1. (**b**) The top 10 significant Gene Ontology Biological Processes (GOBPs) affected by c-Met (MET) and myoferlin (MYOF). Red boxes denote GOBPs enriched for both proteins. FDR, false-discovery rate.

The aforementioned data, which showed a correlation between c-Met and myoferlin expression, were consistent with the functional relationship between myoferlin and various RTKs [5,7,8,9,10]. This suggests that c-Met and myoferlin may be involved together in the progression of ccRCC. To test this hypothesis, we built a network of proteins that interact with both c-Met and myoferlin using STRING and GeneMANIA tools [21,22]. Figure 4a presents the 13 prioritized proteins, consisting of 10 from STRING (blue nodes) and 7 from GeneMANIA (pink nodes) with 4 in common (yellow nodes). These interacting proteins may represent a co-functional module of c-Met and myoferlin. GOBP enrichment analysis of the 13 interacting proteins showed that c-Met and myoferlin were co-enriched for processes related to RTK signaling, which also included CBL, EPS15, PTPN3, KDR, GSK3B, STAT3, CAV1/2, and VEGFA, and muscle development, which also included GSK3B, CAV2, VEGFA, and MYOG (Figure 4b).

### 3.6. HGF and VEGFA Are RTK Ligands That Are Correlated to Myoferlin

**Figure 5:**
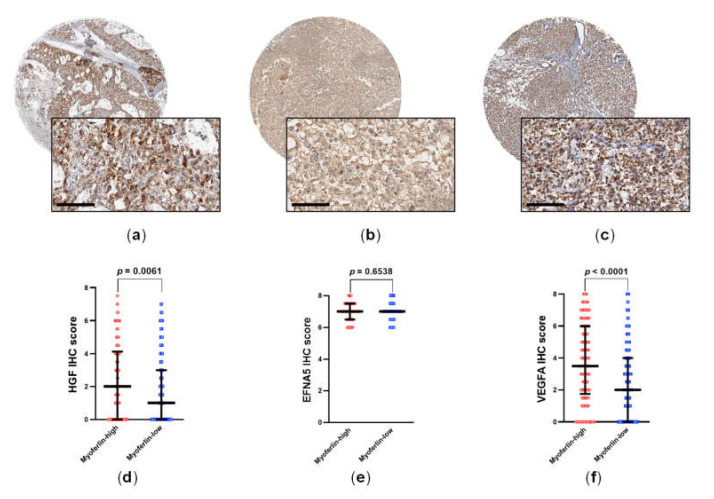
Immunohistochemical (IHC) staining for HGF, EFNA5, and VEGFA and their associations with myoferlin expression in clear cell renal cell carcinoma samples. (**a**) c-Met, (**b**) EFNA5, (**c**) VEGFA, (**d**) Expression of HGF was upregulated in samples with high myoferlin expression, (**e**) expression of EFNA5 and myoferlin showed no correlation, and (**f**) expression of VEGFA was upregulated in samples with high myoferlin expression. Scale bar = 100 μm.

We next examined the expression of RTK ligands, including HGF (Figure 5a), EFNA5 (Figure 5b), and VEGFA (Figure 5c), in the membranes of tumor cells. HGF and EFNA5 are ligands of c-Met and EPHA7, respectively; while VEGFA is a ligand of VEGFR. Notably, VEGFA was also included in the co-functional network for c-Met and myoferlin. HGF and VEGFA levels were significantly upregulated (*p* = 0.0061 and *p* < 0.0001, respectively; Figure 5d,f) and the change in ENFA5 level was not significant (*p* = 0.6538; Figure 5e) in samples with high myoferlin expression, when compared to those with low myoferlin expression.

## 4. Discussion

Modulation of RTK pathways is important for ccRCC treatment, and myoferlin plays pivotal roles in the regulation of various RTKs [25]. LC–MS/MS analysis is suitable for successful discovery of protein biomarkers by directly reflecting the cellular biology and its functional regulators [26,27]. Therefore, using in-depth LC–MS/MS proteomic data, we identified five myoferlin-associated RTK candidates in ccRCC samples, including c-Met, EPHA7, EGFR, HER2, and TEK. A previous study suggested that myoferlin might function as a coactivator of EGFR in ccRCC [11]. The association with myoferlin has not been reported for c-Met and EPHA7. We investigated the correlation of c-Met and EPHA7 with myoferlin using IHC expression and analyzed the prognostic significance in ccRCC. Finally, we focused on the myoferlin-related functions of c-Met through network-based prioritization.

c-Met, a membrane-bound RTK that is constitutively activated by HIF-mediated transcriptional activation, stimulates ccRCC progression [3,28,29,30]. Prior evidence of the independent effect of c-Met expression on ccRCC prognosis has been based on a relatively small number of patients (*n* = 100–200) and only used CSS as an endpoint [31,32]. We showed that c-Met was an independent prognostic factor of OS and CSS in two large cohorts of patients with ccRCC (*n* = 410 and 506). In addition, the present cutoff score for high c-Met expression might be similar to what was used in a previous study that examined the use of c-Met inhibitor cabozantinib in ccRCC treatment, where patients with high c-Met expression (≥2+ staining in 50% of tumor cells) presented survival benefit from receiving cabozantinib therapy compared to the group taking sunitinib, but those without high c-Met expression did not [33]. Thus, our data support the use of IHC staining for c-Met as a prognostic and predictive biomarker in ccRCC.

Through the reconstruction of a protein-interaction network, we also discovered a co-functional network connecting c-Met and myoferlin. This included CBL and CAV1, which have been shown to mediate myoferlin-induced regulation of RTKs [5,9]. Both c-Met and myoferlin have been implicated in myocyte repair and development under physiological conditions [25,34]. GOBP analysis concordantly demonstrated that c-Met and myoferlin were enriched for functions related to the RTK signaling pathway and muscle development. Remarkably, we identified a positive correlation between myoferlin expression and both c-Met and HGF expression in cancer cells, which corroborates a strong connection between myoferlin and the HGF/c-Met pathway in ccRCC. As mesenchymal cells are known to be a predominant source of HGF production, the membranous expression of HGF in cancer cells assessed in this study might represent a paracrine effect on the membrane receptor (i.e., c-Met) [29]. Therefore, this is the first study that infers that myoferlin promotes c-Met expression along with its pro-cancerous functions in ccRCC. These results also support the notion that trifurcate downregulation of myoferlin, c-Met, and HGF could be a potential combination therapy for ccRCC [35].

In addition, the connection between c-Met and myoferlin establishes a potential alternative target for ccRCC treatment, which would counteract resistance to tyrosine kinase inhibitor therapies. Although anti-angiogenic strategies using VEGFR inhibitors, such as sorafenib and sunitinib, have shown considerable effects in ccRCC [3,4], most patients ultimately develop resistance [13,36]. Compensatory activation of the c-Met pathway is a well-known resistance mechanism that provides an alternative signal to angiogenesis and stimulates epithelial–mesenchymal transition [13,37]. Interestingly, myoferlin is also required for angiogenic cascades by maintaining VEGFR2 and TEK expression in endothelial cells [5,7], and VEGFA secretion in pancreatic cancer cells [6]. Consistent with this, the results from this study show that VEGFA expression was significantly associated with myoferlin expression in ccRCC samples. Collectively, it is proposed that inhibition of myoferlin and c-Met could concomitantly regulate the angiogenic switch that evades the suppression of ccRCC by VEGFR inhibition therapy [13].

We also demonstrated that EPHA7 expression is an independent prognostic factor for favorable PFS and CSS in ccRCC, which is a novel finding. Eph receptors are the largest RTK family that influences the behavior of cancers by regulating proliferation, migration, invasion, and angiogenesis [38,39]. We failed to find a correlation between EPHA7 and myoferlin using IHC staining, although this was observed in proteomic analysis. This discrepancy might be ascribed to the site-specific assessment of IHC expression of EPHA7 only in the membrane, excluding the EPHA7 reaction in the cytoplasm. Similar to EPHA7, the expression level of EFNA5, one of the preferred ligands for EPHA7 [39], was not correlated with myoferlin expression. The lack of correlation between myoferlin and both EPHA7 and EFNA5 expression may also represent the distinct regulation of Eph signaling. In contrast to other myoferlin-related RTKs, such as VEGFR2, TEK, and EGFR [5,7,8,9,25], Eph receptors and ephrins relay signals through cell contacts, because they are bound to the cell membrane [39]. In addition, the Eph–ephrin complex undergoes post-activation internalization with the surrounding membrane, which is different from caveolae- or clathrin-dependent endocytosis mediated by myoferlin [38].

Although the current study found that c-Met and myoferlin may have functional connections in ccRCC, the detailed regulatory action was not evaluated. Previous studies have revealed that myoferlin can control RTKs in various ways, including endocytosis, trafficking, recycling, and degradation [25]. Further studies are warranted to define the interaction between c-Met and myoferlin in order to provide alternative therapies for ccRCC, such as dual-targeting treatment.

## 5. Conclusions

In conclusion, c-Met and EPHA7 may be useful biomarkers for prognosis in ccRCC. Targeting the myoferlin-mediated regulation of the c-Met pathway could be a novel therapeutic strategy to enhance c-Met inhibitor treatment and decrease resistance in ccRCC.

## Figures and Tables

**Table 1:** Clinicopathological correlation of immunohistochemical staining for c-Met in clear cell renal cell carcinoma.

Characteristics	Characteristics Category	c-Met High (*n* = 58)	c-Met Low (*n* = 352)	*p*
Age	<58.5	26 (44.8%)	179 (50.9%)	0.4786
	>58.5	32 (55.2%)	173 (49.1%)	
Sex	Male	45 (77.6%)	257 (73.0%)	0.5673
	Female	13 (22.4%)	95 (27.0%)	
Surgery	Radical	44 (75.9%)	228 (64.8%)	0.1321
	Partial	14 (24.1%)	124 (35.2%)	
Body mass index ^1^	<24.4 kg/m^2^	25 (49.0%)	152 (49.2%)	1.0000
	≥24.4 kg/m^2^	26 (51.0%)	157 (50.9%)	
Smoking ^1^	None	43 (84.3%)	250 (80.1%)	0.7002
	Current or past	8 (15.7%)	59 (19.1%)	
Alcohol ^1^	Not heavy	45 (88.2%)	276 (89.3%)	1.0000
	Heavy	6 (11.8%)	33 (10.7%)	
IMDC risk group ^2^	Good/intermediate	36 (85.7%)	246 (96.9%)	0.0058
	Poor	6 (14.3%)	8 (3.1%)	
TNM stage	I or II	37 (63.8%)	283 (80.4%)	0.0078
	III or IV	21 (36.2%)	69 (19.6%)	
WHO grade	1 or 2	8 (13.8%)	185 (52.6%)	<0.0001
	3 or 4	50 (86.2%)	167 (47.4%)	
Myoferlin	Low	22 (37.9%)	265 (75.3%)	<0.0001
	High	36 (62.1%)	87 (24.7%)	

^1^ Lifestyle factors available in 360 patients. ^2^ International Metastatic RCC Database Consortium (IMDC) risk groups available in 296 patients. Abbreviation: TNM, Tumor-Node-Metastasis; WHO, World Health Organization.

**Table 2:** Survival of clear cell renal cell carcinoma patients analyzed by Cox regression including c-Met, TNM stage, and WHO grade.

Survival	Variables	Univariate HR ^1^	*p*	Multivariate HR ^1^	*p*
Progression-free survival	c-Met (high vs. low)	2.516 (1.514–4.183)	0.0004	1.538 (0.9102–2.597)	0.1078
	TNM stage (III/IV vs. I/II)	12.610 (7.896–20.140)	<0.0001	8.729 (5.376–14.174)	<0.0001
	WHO grade (3/4 vs. 1/2)	4.990 (2.841–8.764)	<0.0001	2.440 (1.339–4.446)	0.0036
Overall survival	c-Met (high vs. low)	2.581 (1.652–4.032)	<0.0001	1.834 (1.153–2.919)	0.0105
	TNM stage (III/IV vs. I/II)	5.185 (3.551–7.571)	<0.0001	4.279 (2.840–6.449)	<0.0001
	WHO grade (3/4 vs. 1/2)	2.515 (1.676–3.773)	<0.0001	1.674 (1.043–2.686)	0.0327
Cancer-specific survival	c-Met (high vs. low)	3.811 (2.239–6.485)	<0.0001	1.974 (1.150–3.389)	0.0137
	TNM stage (III/IV vs. I/II)	17.470 (9.719–31.410)	<0.0001	9.813 (5.383–17.886)	<0.0001
	WHO grade (3/4 vs. 1/2)	15.570 (5.644–42.940)	<0.0001	6.200 (2.171–17.706)	<0.0001

^1^ Hazard ratio (HR) with 95% confidence interval. Abbreviation: TNM, Tumor-Node-Metastasis; WHO, World Health Organization

**Table 3:** Validation of the prognostic effect of c-Met expression in an independent cohort of clear cell renal cell carcinoma.

Survival	Variables	Univariate HR ^1^	*p*	Multivariate HR ^1^	*p*
Overall survival	c-Met (High vs. Low)	3.674 (2.370–5.698)	<0.0001	2.381 (1.503–3.771)	0.0002
	TNM stage (III/IV vs. I/II)	5.892 (4.071–8.527)	<0.0001	4.492 (2.889–6.985)	<0.0001
	WHO grade (3/4 vs. 1/2)	2.928 (2.007–4.271)	<0.0001	1.331 (0.840–2.111)	0.2236

^1^ Hazard ratio (HR) with 95% confidence interval. Abbreviation: TNM, Tumor-Node-Metastasis; WHO, World Health Organization

**Table 4:** Survival of clear cell renal cell carcinoma patients analyzed by Cox regression including EPHA7, TNM stage, and WHO grade.

Survival	Variables	Univariate HR ^1^	*p*	Multivariate HR ^1^	*p*
Progression-free survival	EPHA7 (Low vs. High)	2.511 (1.358–4.644)	0.0033	2.311 (1.237–4.319)	0.0086
	TNM stage (III/IV vs. I/II)	11.368 (7.118–18.156)	<0.0001	9.277 (5.710–15.074)	<0.0001
	WHO grade (3/4 vs. 1/2)	4.585 (2.610–8.054)	<0.0001	2.485 (1.377–4.484)	0.0025
Overall survival	EPHA7 (Low vs. High)	1.814 (0.993–3.315)	0.0528	1.655 (0.898–3.050)	0.1063
	TNM stage (III/IV vs. I/II)	5.342 (3.617–7.892)	<0.0001	4.527 (3.007–6.813)	<0.0001
	WHO grade (3/4 vs. 1/2)	2.773 (1.795–4.286)	<0.0001	1.824 (1.151–2.891)	0.0105
Cancer-specific survival	EPHA7 (Low vs. High)	2.979 (1.548–5.733)	0.0011	2.352 (1.214–4.558)	0.0113
	TNM stage (III/IV vs. I/II)	15.906 (8.846–28.601)	<0.0001	10.712 (5.879–19.516)	<0.0001
	WHO grade (3/4 vs. 1/2)	14.460 (5.242–39.889)	<0.0001	6.680 (2.362–18.888)	0.0003

^1^ Hazard ratio (HR) with 95% confidence interval. Abbreviation: TNM, Tumor-Node-Metastasis; WHO, World Health Organization

## Data Availability

The data presented in this study are available on request from the corresponding author. The data are not publicly available due to privacy restrictions.

## Supplementary Materials

The following are available online at https://www.mdpi.com/article/10.3390/cancers14041095/s1, Table S1: Ongoing clinical trials for receptor tyrosine kinase inhibitors in renal cell carcinomas. Table S2: Receptor tyrosine kinase proteins and their correlation with myoferlin in the clear cell renal cell carcinoma proteomic dataset. Figure S1: Selected non-RTK proteins identified in the ccRCC proteomic dataset and their correlation with myoferlin.
