# Supplementary material for: c-Met and EPHA7 Receptor Tyrosine Kinases Are Related to Prognosis in Clear Cell Renal Cell Carcinoma: Focusing on the Association with Myoferlin Expression
Source: Cancers (Basel). 2022 Feb 21;14(4):1095. doi: 10.3390/cancers14041095 (PMC8870418; doi:10.3390/cancers14041095)
Supplement: Supplementary file 1 [file cancers-14-01095-s001.zip › Figure S1_Selected non-RTK proteins identified in the ccRCC proteomic dataset and their correlation with myoferlin_1.0.pdf]

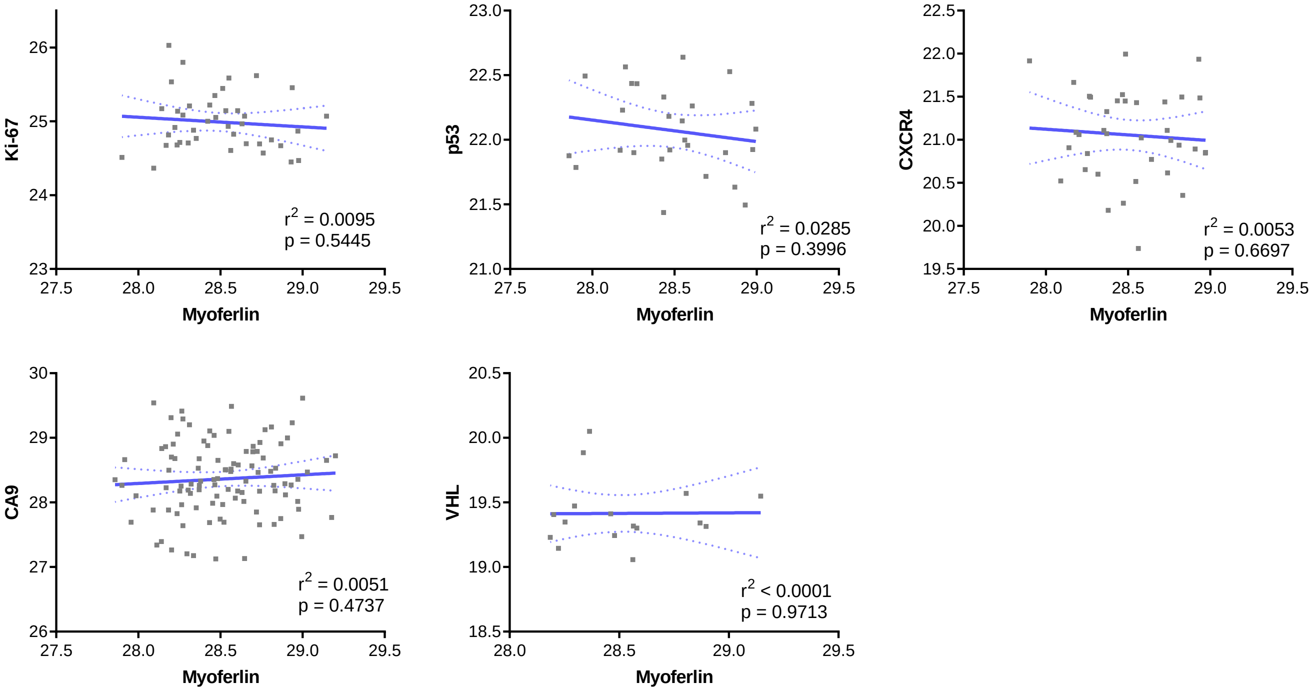

Figure S1. Selected non-RTK proteins identified in the ccRCC proteomic dataset and their correlation with myoferlin. Dotted lines denote the 95% confidence interval of Pearson′s correlation.
